# Application of spatial transcriptomics analysis using the Visium system for the mouse nasal cavity after intranasal vaccination

**DOI:** 10.3389/fimmu.2023.1209945

**Published:** 2023-07-21

**Authors:** Sakiko Toyama, Tomoko Honda, Sadahiro Iwabuchi, Shinichi Hashimoto, Kenzaburo Yamaji, Yuko Tokunaga, Yusuke Matsumoto, Hideya Kawaji, Takashi Miyazaki, Yoshiaki Kikkawa, Michinori Kohara

**Affiliations:** ^1^ Department of Microbiology and Cell Biology, Tokyo Metropolitan Institute of Medical Science, Tokyo, Japan; ^2^ Graduate School of Medical and Dental Sciences, Niigata University, Niigata, Japan; ^3^ Department of Molecular Pathophysiology, Institute of Advanced Medicine, Wakayama Medical University, Wakayama, Japan; ^4^ Transboundary Animal Diseases Research Center, Joint Faculty of Veterinary Medicine, Kagoshima University, Kagoshima, Japan; ^5^ Research Center for Genome and Medical Sciences, Tokyo Metropolitan Institute of Medical Science, Tokyo, Japan; ^6^ Business Management Department, Toko Yakuhin Kogyo Co., Ltd., Toyama, Japan; ^7^ Deafness Project, Tokyo Metropolitan Institute of Medical Science, Tokyo, Japan

**Keywords:** carboxyvinyl polymer (CVP), intranasal vaccine, nasal passage (NP), spatial transcriptomics, Visium, microfold (M) cell

## Abstract

Intranasal vaccines that elicit mucosal immunity are deemed effective against respiratory tract infections such as severe acute respiratory syndrome coronavirus 2 (SARS-CoV-2), but their ability to induce humoral immunity characterized by immunoglobulin A (IgA) and IgG production is low. It has been reported that vaccination with a mixture of a viscous base carboxyvinyl polymer (CVP) and viral antigens induced robust systemic and mucosal immune responses. In this study, we analyzed the behavior of immunocompetent cells in the nasal cavity over time by spatial transcriptome profiling induced immediately after antigen vaccination using CVP. We established a method for performing spatial transcriptomics using the Visium system in the mouse nasal cavity and analyzed gene expression profiles within the nasal cavity after intranasal vaccination. Glycoprotein 2 (*Gp2*)*-*, SRY-box transcription factor 8 (*Sox8*)*-*, or Spi-B transcription factor (*Spib*)-expressing cells were increased in the nasal passage (NP) region at 3–6 hr after SARS-CoV-2 spike protein and CVP (S-CVP) vaccination. The results suggested that microfold (M) cells are activated within a short period of time (3–6 hr). Subsequent cluster analysis of cells in the nasal cavity showed an increase in Cluster 9 at 3–6 hr after intranasal vaccination with the S-CVP. We found that *Il6* in Cluster 9 had the highest log2 fold values within the NP at 3–6 hr. A search for gene expression patterns similar to that of *Il6* revealed that the log2 fold values of *Edn2*, *Ccl20*, and *Hk2* also increased in the nasal cavity after 3–6 hr. The results showed that the early response of immune cells occurred immediately after intranasal vaccination. In this study, we identified changes in gene expression that contribute to the activation of M cells and immunocompetent cells after intranasal vaccination of mice with antigen-CVP using a time-series analysis of spatial transcriptomics data. The results facilitated the identification of the cell types that are activated during the initial induction of nasal mucosal immunity.

## Introduction

In recent years, severe acute respiratory syndrome coronavirus-2 (SARS-CoV-2), which causes coronavirus disease (COVID-19), has spread worldwide and become a serious public health problem. A variety of vaccines against SARS-CoV-2 have been developed and administered, typically, intramuscularly or subcutaneously. However, the main route of infection by SARS-CoV-2 is through the upper respiratory tract, and a vaccine with a protective effect at the site of infection is desired. In contrast to intramuscular vaccination, mucosal immunization is a non-invasive procedure that induces a strong and long-lasting secretory immunoglobulin A (IgA) response and plays an important role in defense against viral infection of the upper respiratory tract (1–3). Therefore, it is important to study mucosal immunity, especially nasal immunity, which has been shown to play an important role in IgA production, mainly in nasal-associated lymphoid tissue (NALT) and Waldeyer’s tonsillar ring (4–6), which appears to be functionally equivalent to NALT in rodents (7). Furthermore, both microfold (M) cells, which are known to play an important role in initiating local immune responses in mucosal tissues, and dendritic cells (DCs), which are antigen-presenting cells, in nasal passages (NP) play a major role in mucosal immune activation (8). Although effective and safe adjuvants are needed for intranasal vaccines, none have yet been approved for use (9–12). Previous studies have reported that intranasal inoculation with a mixture of carboxyvinyl polymer (CVP) and antigen strongly enhances the production of IgG and IgA antibodies in blood, bronchoalveolar lavage fluid (BALF), and nasal lavage fluid against influenza and hepatitis B viruses (13, 14). The viscosity of the mucoadhesive CVP has been demonstrated to increase the mucosal and systemic immune response (13). Based on these reports, we administered the SARS-CoV-2 spike protein and CVP (S-CVP) intranasally to mice. The results showed that antibody titers increased markedly compared to vaccination with spike protein alone and protected mice against COVID-19 symptoms more effectively than when conventional adjuvant (Alum) was used (15). However, it is unclear where in the nasal cavity the S-CVP vaccine is most effective for inducing immunity because the nature of the cellular response and cellular activity in the nasal cavity have not yet been clarified.

Immunohistochemical staining, fluorescence activated cell sorter (FACS), and *in situ* methods have been used to identify the different types of immune cells and to analyze the activation status of cells in tissues. However, these methods can only identify a limited number of cell types and/or genes and have limited sensitivity (16). Therefore, we decided to apply spatial transcriptome (ST) analysis to solve these problems.

Using an ST method that employs PCR amplification and next generation sequencing (NGS), individual transcripts can be detected with high sensitivity and original location information can be determined using unique positional molecular barcodes (17). Such positional information cannot be obtained by conventional FACS or single cell analysis. While the ST method can detect genes with high sensitivity and comprehensiveness using PCR, it has the disadvantage of low resolution. Conversely, multiplex immunofluorescence methods are capable of high-resolution analyses at the single-cell level; however, they can analyze a limited number of genes. Further, by analyzing the changes in this information over time, it is possible to accurately clarify cellular and genetic responses within tissues. In other words, ST analysis can reveal which types of cells are present, and when and where they are present. The ability to comprehensively analyze spatial gene expression in the NP after intranasal CVP vaccination may lead to the discovery of as yet unknown immune responses to vaccination. In this study, we used the Visium system produced by 10x Genomics to perform ST analysis of the nasal cavity after intranasal CVP vaccination. This technique measures the complete transcriptome at a high spatial resolution using barcoded oligonucleotides that are arranged in arrays of up to 5,000 spots measuring 55 µm in diameter. Therefore, in order to obtain the complete transcriptome of the nasal cavity at a high spatial resolution after nasal vaccination, we sought to establish a method to perform ST analysis using the Visium system in the nasal cavity. Because the mouse nasal cavity contains bones and cartilage, it is necessary to decalcify the tissues in order to obtain thin sections of nasal tissues while preserving their structure. Decalcification is a procedure that removes calcium and softens hard tissues such as bones and teeth so that they can be cut into thin sections. The standard decalcification procedure for histopathology involves immersing the tissue in a decalcifying solution at room temperature or at elevated temperatures for an extended period of time (3–7 days, depending on the type of tissue). However, in this study, such long incubation periods adversely affected the extraction of sufficient qualities of RNA for ST analysis. To date, there have been no reports on decalcified tissue for ST analysis using the Visium system. It is therefore necessary to develop a protocol for performing ST analysis using the Visium system with decalcified nasal cavity tissue in which the RNA has not been degraded. Here, we clarified the optimal decalcification period for performing ST analysis and then conducted ST analysis using the Visium system on nasal tissues.

## Materials and methods

### Ethics statement

This study was carried out following the Guidelines for Animal Experimentation of the Japanese Association for Laboratory Animal Science and the recommendations set out in the Guide for the Care and Use of Laboratory Animals published by the National Institutes of Health. All protocols were approved by the Tokyo Metropolitan Institute of Medical Science Animal Experimental Committee (Approval No. 22-078).

### Animals

In all experiments, 5–6-week-old BALB/c mice were purchased from Japan SLC (Shizuoka, Japan). The mice were given free access to rodent chow and water, and were maintained on a 12 hr light/12 hr dark cycle. In our previous study, we administered the SARS-CoV-2 spike protein and carboxyvinyl polymer (CVP) (Toko Yakuhin Kogyo Co., Ltd., Toyama, Japan) (S-CVP) intranasally to mice (15). To ensure that the results were comparable with those of the previous study, only female mice were used in this study.

### Immunogen

A purified His-tagged active trimer of the SARS-CoV-2 S protein (#SPN-C52H8, Wuhan-Hu-1[R683A, R685A], Newark, DE) was purchased from AcroBiosystems (Newark, DE).

### Vaccination of S-CVP

Mice were administered with 5 μg of the SARS-CoV-2 spike protein and CVP (S-CVP). The immunized mice were immunized at 3, 6, and 24 hr. The non-immunized mice that had not been administered the S-CVP were considered the healthy control mice (i.e., 0-hr mice). The CVP was mixed with an equal volume of protein solution just before vaccination. Mice were then immunized intranasally by 10 μL intranasal instillation using a microsyringe (5 μL in each NP) (#MS-NE10, Ito Corporation, Shizuoka, Japan). Mice were immunized once, euthanized at 3, 6, and 24 hr after immunization, and their heads were collected for tissue analysis.

### Formalin-fixed paraffin-embedded block production

Mice under anesthesia were dissected, and each head was separated into right and left halves using a scalpel. Tissues were fixed by immersing in 4% paraformaldehyde (PFA) (#315-1; TAAB, Berkshire, UK)/PBS(-) pH 7.0–8.0 at RT for 24 hr. After washing with Milli-Q water (18.2 MΩ.cm) obtained from a Millipore Milli-Q Integral 5 water purification system (Merck, Darmstadt, Germany), the tissues were immersed in decalcification solution (10% EDTA-4Na (#347-01881; FUJIFILM Wako, Osaka, Japan)/PBS(-) with 1.5% citric acid monohydrate (#09106-15; Nacalai Tesque, Kyoto, Japan) pH 7.0–7.5) and subjected to continuous shaking at RT for 46–50 hr to promote decalcification. The tissues were placed on grates in a container to ensure that they did not touch the bottom. After washing with Milli-Q water, the decalcified heads were penetrated with paraffin for 20 hr and then embedded in paraffin. Tissue-Tek Paraffin Wax II (#No. 7810; Sakura Finetek Japan, Tokyo, Japan) and Paraffin (#164-13345; FUJIFILM Wako) were used for tissue penetration and embedding; they were mixed at 25:2.1 for penetration or 25:4.2 for embedding. The prepared FFPE blocks were stored at 4°C until thin sectioning was performed. A microtome (#REM-710; Yamato Kohki, Saitama, Japan) and blades (#S35, FEATHER Safety Razor, Osaka, Japan) were used to prepare tissue slices.

### Check of RNA quality for ST analysis

For RNA extraction from the FFPE blocks of mouse heads containing the NP and NALT, 8 slices (10 µm) from a region were pooled per specimen (approx. 5 mm × 5 mm). Genomic RNA was isolated using a RNeasy FFPE Kit (#73504, QIAGEN, Hilden, Germany) according to the manufacturer’s instructions. Total RNA was eluted with 20 μL of RNase-free water. RNA concentration was determined using a NanoPhotometer NP80 (Implen, Munich, Germany) and the average fragment size was estimated using an Agilent Bioanalyzer (RNA 6000 Nano Kit, #5067-1511, Agilent, Waldbronn, Germany). Instead of an RNA integrity number, Visium Spatial for FFPE (#100339, #100194, #100251, 10x Genomics Inc., Pleasanton, CA) uses a DV200 value as indicator of RNA quality. According to the manufacturer, DV200 values of > 50% are recommended as being suitable for analysis.

### Library preparation and sequencing of the ST analysis

ST analysis using the Visium system was performed according to the manufacturer’s instructions. Briefly, mouse FFPE samples were sectioned to a thickness of 5 μm. The sections were placed on Visium slides and ordered as a time series within the capture area (6.5 × 6.5 mm). Images of hematoxylin and eosin-stained sections were obtained after staining with HE. HE staining and imaging for Visium analysis were performed according to the Visium Spatial Gene Expression for FFPE User Guide (CG000409 Rev A, 10x Genomics Inc.). Image data acquisition was performed using an inverted fluorescence phase contrast microscope (BZ-X710, KEYENCE, Tokyo, Japan). Libraries for Visium were prepared according to the Visium Spatial Gene Expression for FFPE User Guide (CG000407 Rev C, 10x Genomics Inc.). The quality and quantity of libraries were measured using an Agilent 4200 TapeStation Qbit Fluorometer (Thermo Fisher Scientific, Waltham, MA) and a KAPA Library Quantification kit (#KK4824, Roche, Basel, Switzerland). The average size and concentration of libraries was 233–239 bp and 2.1–4.42 nM, respectively. Libraries were sequenced on a NextSeq 550 System (Illumina, San Diego, CA) using a NextSeq 500/550 High Output Kit v2.5 (150 cycles) (#20024907, Illumina) at a depth of approximately 69–127 million reads per sample.

### Hematoxylin and eosin staining for ST

Hematoxylin and eosin (HE) staining and imaging for Visium analysis were performed according to the Visium Spatial Gene Expression for FFPE User Guide (CG000409 Rev A; 10x Genomics Inc.). Briefly, the slides with tissue sections were placed on a thermocycler adaptor with the active surface facing up and incubated for 2 hr at 60°C. The slides were removed from the thermal cycler and allowed to cool to room temperature (RT). The tissue sections were deparaffinized in xylene two times for 10 min and then rehydrated twice in 100% ethanol for 3 min. The slides were placed on the thermocycler adaptor with the active surface facing up and incubated at 37°C for 15 min. The slides were then immersed in 96% ethanol for 3 min, 70% ethanol for 3 min, and Milli-Q water for 20 sec. Thereafter, the slides were immersed in Mayer’s Hematoxylin (#S330930-2; Agilent) at RT for 3 min. They were then immersed in a bluing buffer (#CS70230-2; Agilent) at RT for 1 min and incubated with alcoholic eosin (#HT110116; Merck) at RT for 1 min. Glycerol (85%) was added on the tissue sections, which were then covered with coverslips.

### Data analysis and visualization of the ST

To integrate the four data sets, raw FASTQ files and histology images were processed using the aggr pipeline implemented in Space Ranger-1.3.1 (10x Genomics Inc.). The number of spots under tissues in unvaccinated mice or in mice at 3, 6, 24 hr after vaccination was 2,069, 1,967, 2,235, and 1,889 spots respectively, where the total number of spots arrayed on a single slide of the Visium platform is 5,000. The mean number of reads per spot was 52,872 and 63,343, 56,836, and 36,628, respectively. The median of the number of genes per spot was 6,545 and 6,179, 6,686, and 5,597, respectively.

The results were processed using Loupe Browser 6.1.0 (10x Genomics Inc.). Spots corresponding to bone in the HE-stained slides were selected manually. The gene expression data for the graph-based clusters generated by the Space Ranger software were exported as CSV files. The data consisted of the median-normalized average of gene expression, log2 fold changes, and statistical significance (p values) computed by the “Globally Distinguishing” method for genes with a p value < 0.05 in any of the clusters. The 25 most expressed genes with p values < 0.05 in the csv file were sorted in order of increasing “Median Normalized Average”.

Spots in the HE slides that corresponded to bone were selected in Loupe Browser 6, colored red, and saved as “Bone” and as same as “Nasal epithelium and Lymphoid tissues” (this image will henceforth be referred to as the dot pattern). The “Significant Feature Comparison” setting was set to “Globally Distinguishing” and “Feature Type” was set to “Gene”. The list of genes expressed in the selected region was extracted and the “Displayed Numeric Value” in the “Feature Table Options” was set to the “Median-Normalized Average”. “CSV Export Count” was set to “All Gene Expression” and the.csv file was prepared. The 25 most expressed genes with p values < 0.05 in the csv file were sorted in order of increasing “Median Normalized Average”. It is important to note that, compared to other software such as SEURAT, the analyses performed using Space Ranger and Loupe Browser are limited to filtering, integration, and dimensionality reduction.

### Nasal histopathology and immunohistochemistry

The mice nasal samples were fixed in 10% neutral buffered formalin, embedded in paraffin, and sectioned at a thickness of 4 μm for histological analysis. For immunohistochemical (IHC) evaluation, deparaffinized tissue sections were treated by autoclaving sections in 10 mM citrate buffer (pH 6.0) for 10 minutes at 121°C for antigen retrieval. The sections were then immersed in 1% hydrogen peroxide in methanol at room temperature for 20 min to inactivate endogenous peroxidase. The sections for CD4 and CD8α staining were blocked with BlockAce (#UKB80, DS Pharma Biomedical, Osaka, Japan) for 20 min at room temperature. They were then incubated overnight at 4°C with CD4-specific antibody (#ab183685, [EPR19514], 1:500, Abcam, Cambridge, UK) or CD8 alpha-specific antibody (#98941, [D4W2Z], 1:500, Cell Signaling Technology, Danvers, USA) diluted in 0.25% Tween20 PBS. The sections for CD19 staining was blocked with Odyssey blocking buffer (#927-40000, LI-COR, Lincoln, NE) for 20 min at room temperature. Samples were then incubated overnight at 4°C with CD19 specific antibody (#90176, [D4V4B] 1:8000, Cell Signaling Technology, Danvers, USA) diluted with Can Get Signal Immunostain Immunoreaction enhancer solution B (#NKB-601, TOYOBO, Osaka, Japan). Subsequently, secondary labeling was performed by incubation with EnVision+ System-HRP-labeled Polymer Anti-Rabbit (#K4003, Dako Denmark A/S, Glostrup, Denmark) for 1 hr at room temperature. Detection with DAB chromogen was completed using an ImmPACT DAB Peroxidase Substrate kit (#SK−4105, Vector Laboratories, Newark, CA, USA). Nuclear staining was performed with hematoxylin solution.

### FACS analysis

NALT and NP cells from mice were collected under a microscope (Stemi 305 Cam, Carl Zeiss, Oberkochen, Germany). The cells were then stained with fluorescence-conjugated antibodies for cell surface markers for 20 min at 4°C. The following fluorescence-conjugated monoclonal antibodies were used for FACS analysis: APC-conjugated anti-mouse CD3e antibody (#17-0031-82, eBioscience, San Diego, California), APC-Cyanine (Cy)7-conjugated anti-mouse CD4 antibody (#100413, BioLegend, San Diego, California), Pacific Blue-conjugated anti-mouse CD8a antibody (#100728, BioLegend), Brilliant Violet (BV)650-conjugated anti-mouse CD11c antibody (#117339, BioLegend), phycoerythrin (PE)-CF594-conjugated anti-mouse CD19 antibody (#562291, BD Biosciences, San Jose, California), PE-Cy7-conjugated anti-mouse CD45 antibody (#103113, BioLegend), and BV605-conjugated anti-mouse F4/80 (#123133, BioLegend). Cells were fixed in 2.5% formalin/PBS(-) before being resuspended in 1 mL FACS buffer for analysis using a flow cytometer system (BD LSRFortessa™ X-20, BD Biosciences).

### Volcano plot

A volcano-plot was created using the R statistical software package (version 4.2.2, R Core Team (2022); Vienna, Austria) and RStudio (version 2022.12.0, Build 353, RStudio Inc., Boston, Massachusetts).

## Results

### Establishment of a protocol for obtaining an ST analysis of mouse nasal cavity tissues

First, we attempted to establish a protocol for obtaining thin slices of the mouse nasal cavity that contained enough quality RNA for ST analysis. This is a challenging step because slicing the mouse nasal tissue requires pre-processing by decalcification, which typically damages RNA. Using a two-day decalcification step with a solution containing both EDTA and citric acid, we established a protocol that allowed us to obtain the sliced sections while limiting damage to the RNA (i.e., DV200 values of above 70%) (**Supplemental Figure 1A**).

By following the manufacturer’s instructions using FFPE blocks prepared from BALB/c mouse nasal tissue, we performed ST analysis using the Visium system. Briefly, 5 μm sections of FFPE samples were placed on Visium slides and ordered as a time series within the capture area (6.5 × 6.5 mm). Images of HE-stained sections were obtained after staining with HE. NGS libraries were subsequently prepared by synthesizing cDNA on the glass slides. The resulting NGS library was sequenced and analyzed using Space Ranger and Loupe Browser 6 software from 10x Genomics (**Supplemental Figure 1B**).

Based on morphological evaluation of the HE-stained slides, “bone” and “nasal epithelium and lymphoid tissue” were selected for analysis as these features were the easiest to evaluate morphologically. HE-stained tissue images showed the frontal bone in the upper cranium, the nasal passage, a hematoxylin-stained area in the nasal cavity, and NALT in the nasopharynx. We wanted to ascertain whether the genes in bone could be identified after demineralization, and whether the genes in lymphocytes could be identified in the lymphoid tissue, as we were interested in clarifying immune system dynamics in the lymphoid tissue.

**Figure 1:**
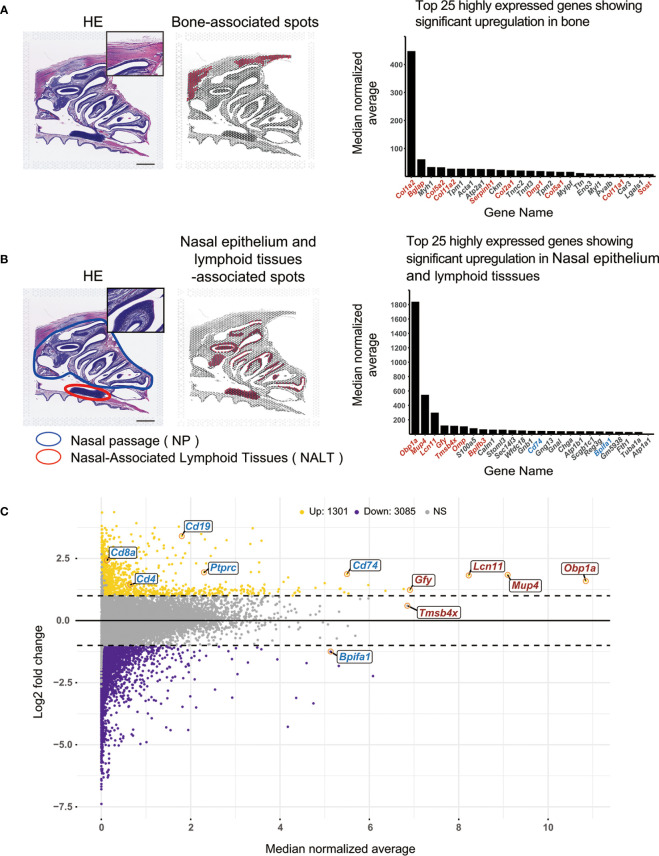
Application of the Visium system to assay gene expression in mouse nasal tissues. **(A)** Spatial transcriptome analysis using the Visium system in bone tissue Left: HE staining of a mouse head in cross-section. Middle: Spots within the area determined to be bone areas based on morphological evaluation of the HE-stained tissue are shown in red. Scale bar = 1 mm. Right: The 25 most expressed genes in the selected spots are shown in the bar graph. Red letters indicate genes related to bone or supporting muscle. Black letters indicate other genes. The bar graph was created using the R statistical software package (version 4.2.2). **(B)** Spatial transcriptome analysis of the nasal epithelium and lymphoid tissue area using the Visium system. Left: HE staining of a mouse head in cross-section. Scale bar = 1 mm. Middle: Spots within areas that have been morphologically evaluated based on HE-stained tissue sections and determined to contain lymphocytes are shown in red. Right: The 25 most expressed genes in the selected spots are shown in bar graph. Red letters indicate genes that are related to nasal functions (e.g., olfaction). Blue letters indicate genes related to the immune system. Black letters indicate other genes. The bar graph was created using the R statistical software package (version 4.2.2). **(C)** Chart of areas that have been morphologically evaluated based on HE-stained tissue sections and determined to contain lymphocytes. A total of 18,404 genes were identified as being expressed in nasal epithelium and lymphoid tissue-associated spots based on log2 fold changes and median normalized average. The chart was created using the R statistical software package (version 4.2.2). Horizontal axis: Median normalized average of gene expression levels for each selected spot. Vertical axis: Log2 fold changes in gene expression levels for selected spots. Log2 fold changes ≥ 1 are indicated by yellow dots, log2 fold changes ≤-1 are indicated by violet dots, and all others are indicated by gray dots. Averages of the top five most expressed genes and genes related to olfactory function are indicated by red letters; genes related to immune function are indicated by blue letters. Up, upregulated; Down, downregulated; NS, not significant.

We manually selected two sets of spots on HE-stained slides; each set corresponded to “bone” and “nasal epithelium and lymphoid tissue” (214 and 411 spots, respectively). We then checked the gene expression profiles of these spots in the naïve mouse sample (**Figure 1A**). Of the 25 most highly expressed genes showing significant up-regulation (p values < 0.05) in the bone-associated spots, 10 were specifically related to bone and muscle. The most expressed gene, *Col1a2*, encodes type I collagen, the main component of connective tissue, and mutations in the *Col1a2* gene are known to cause osteogenesis imperfecta (18). The second most expressed gene, *Bglap*, encodes the osteogenic marker osteocalcin, a non-collagenous protein that is most abundant in the bone matrix and is a bone-derived endocrine hormone (19). Other genes related to bone and muscle were *Col5a2*, *Col11a2*, *Serpinh1*, *Col2a1*, *Dmp1*, *Col5a1*, *Col11a1*, and *Sost*.

The area circled in blue on the HE-stained image (**Figure 1B**) is the NP, and the area circled in red is the NALT. Of the 25 most highly expressed genes showing significant up-regulation (p values < 0.05) in the nasal epithelium and lymphoid tissue-associated spots, most were found to be related to nasal function (olfaction) or to the connective tissues that make up the nose. The gene for Odorant-binding protein 1a (*Obp1a*), the most abundant gene in the nasal epithelium and lymphoid tissue, encodes an odorant-binding protein and is considered to mediate olfactory transduction in mice (20). Among the olfactory system proteins that function to reversibly bind to odors and pheromones, *Mup4* encodes Major urinary protein 4, an isoform of major urinary proteins (MUPs) (21–23). MUPs are expressed at pheromone excretion sites (liver and kidney), while MUP4 is the nasal isoform (24). Of the 25 most highly expressed genes, seven were related to nasal functions (red) (**Figure 1B**) and two were involved in immunity, i.e., *Cd74* and *Bpifa1* (blue) (**Figure 1B**). We then compared all 18,404 genes expressed in the nasal epithelium and lymphoid tissue-associated spots using log2 fold changes (vertical axis) and median normalized average (horizontal axis) (**Figure 1C**). The five most highly expressed genes (*Obp1a, Mup4, Lcn11, Gfy*, and *Tmsb4x*) and six nasal immunity-related genes (*Cd74*, *Bpifa1*, *Ptprc* (CD45), *Cd19*, *Cd4*, and *Cd8a*) (25) are shown in red and blue, respectively (**Figure 1C**). Visium effectively detected genes that are highly expressed in the nasal cavity, such as those in the supporting tissues of the nasal cavity, as well as genes that were weakly expressed (i.e., micro gene expression), such as *Cd8a*. Log2 fold changes in *Ptprc*, *Cd19*, *Cd4*, and *Cd8a* were all ≥ 1, indicating that expression was higher in the red spots of the selected area than the median normalized average for the overall region. The median normalized average gene expression levels were used to identify *Ptprc*, *Cd19*, *Cd4*, and *Cd8a* using the Visium system (**Figure 1C**). The immunohistochemical analysis also detected CD19, CD4, and CD8a positive cells in NALT and NP (**Supplemental Figure 2**). The FACS analysis also detected CD45, CD19, CD4 and CD8 positive cells in the NALT and NP (**Supplemental Figure 3**).

### Spatial gene expression profiles after intranasal vaccination

**Figure 2:**
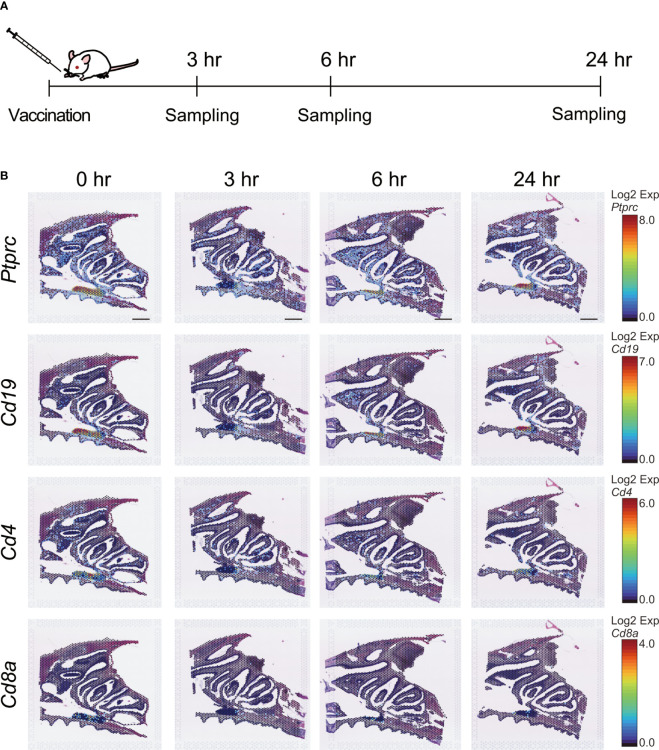
Immunocompetent cells related to gene expression after intranasal vaccination. **(A)** Experimental schematic. Six-week-old female BALB/c mice in the treatment group were immunized via the intranasal route with an S-CVP intranasal vaccine. Mice in the non-treated group were defined as 0 hr. Mice in the treated group were euthanized at 3, 6, and 24 hr after treatment and their heads were sampled. Cross-sections of mouse heads were utilized for spatial transcriptome analysis. **(B)** Spatiotemporal changes in *Ptprc*, *Cd19*, *Cd4*, and *Cd8a* expression in mouse nasal cavity at 0, 3, 6, and 24 hr. Scale bar = 1 mm.

We then used ST analysis to evaluate the effect of S-CVP intranasal vaccination on mice. To identify the genes involved in the induction of immune responses by the S-CVP vaccination, BALB/c mice were administered the S-CVP intranasal vaccine. Mouse heads were sampled at 0 hr (unvaccinated) and at 3, 6, and 24 hr post-vaccination (**Figure 2A**). Changes in *Ptprc*, *Cd4*, *Cd8a*, and *Cd19*, which are genes that are representative of the immune response, were also examined (**Figure 2B**). The expression of *Ptprc* (Protein tyrosine phosphatase receptor type C, or CD45), which was originally considered to be a leukocyte common antigen, can be used as a marker for all differentiated hematopoietic cells. In this study, the expression of *Ptprc* was observed in the mouse nasal tissues throughout the experimental period (i.e., at 0, 3, 6 and 24 hr post-vaccination) (**Figure 2B**). A similar pattern of gene expression was observed in other specific lymphoid cell makers, such as *Cd19* (B-lymphocyte surface antigen), *Cd4* (a glycoprotein that serves as a co-receptor for the T-cell receptor), and *Cd8a* (a glycoprotein found on most cytotoxic T cells) (**Figure 2B**). ST analysis using the Visium system revealed that in non-vaccinated animals, cells associated with the immune response are present in the nasal cavity, including the NP and NALT. In addition, gene expression of *Ptprc*, *Cd19*, *Cd4*, and *Cd8a* was not significantly altered shortly after S-CVP vaccination.

**Figure 3:**
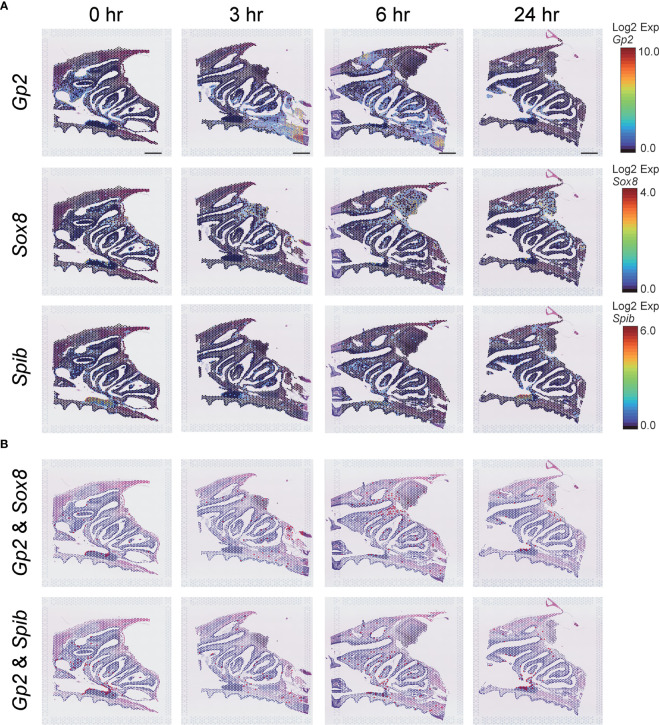
M-cell-related gene expression analysis estimated using the Visium system. **(A)** Spatiotemporal changes in *Gp2*, *Sox8*, and *Spib* expression in mouse nasal cavity at 0, 3, 6, and 24 hr. Scale bar = 1 mm. **(B)** Merged images of *Gp2* and *Sox8*, *Gp2* and *Spib* expression in the mouse nasal cavity at 0, 3, 6, and 24 hr.

M cells, which are known to play an important role in initiating local immune responses in mucosal tissues(8), have been detected in the follicle-associated epithelium of Peyer’s patches in the villous epithelium of the small intestine and the nasal cavity. *Gp2* (Glycoprotein 2), *Sox8* (SRY-box transcription factor 8), and *Spib* (Ets transcription factor Spi-B) are M cell markers (26–29), and data obtained from ST analysis of the nasal cavity after S-CVP vaccination were used to examine changes in these M cell markers at 0, 3, 6, and 24 hr (**Figures 3A, B**). The expression of the M-cell marker genes was observed to increase in the NP at 3–6 hr after S-CVP vaccination (**Figure 3A**). In addition, gene expression patterns of *Gp2* & *Sox8* and *Gp2* & *Spib* were altered shortly after administering the S-CVP vaccination, i.e., at 3–6 hr (**Figure 3B**).

### Changes in the gene expression profiles of the clusters after vaccination

**Figure 4:**
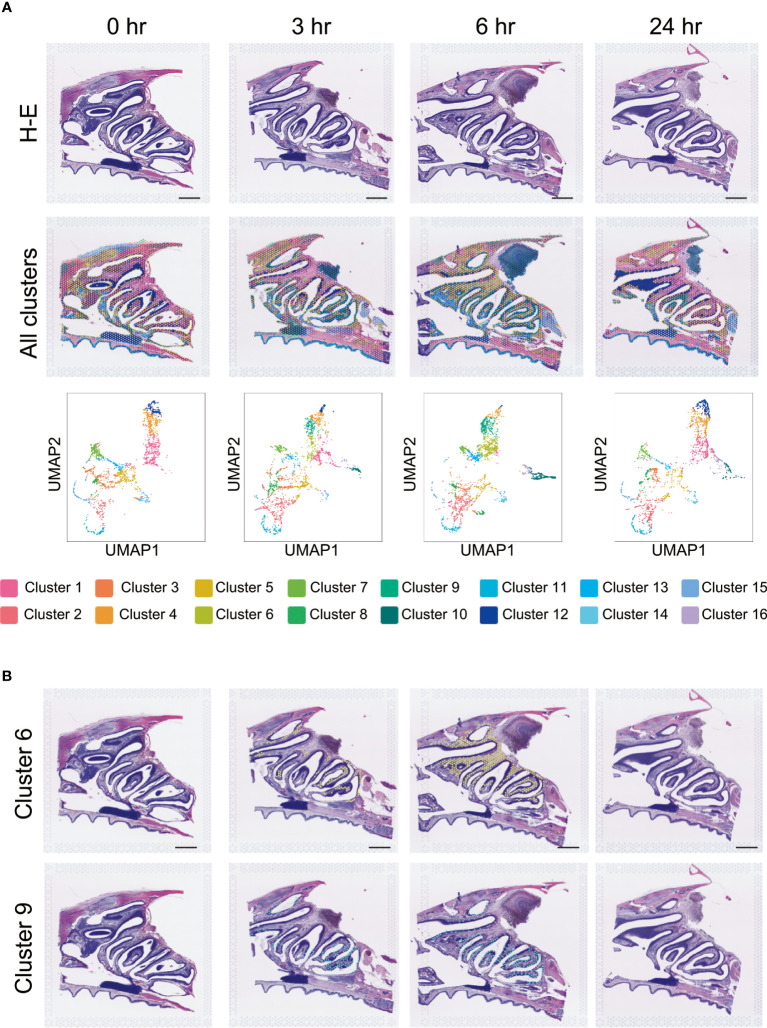
Cluster analysis after S-CVP intranasal vaccination. **(A)** Cluster analysis after S-CVP intranasal vaccination. Visium data of the regions of the HE-stained section were analyzed using the Loupe Browser 6 program; the data were divided into 16 clusters by the analysis. Visualization of the spots after dimension reduction by UMAP analysis. Scale bar = 1 mm. **(B)** Expression analysis of Clusters 6 and 9 over time. Both clusters show a characteristic pattern of non-expression at 0 hr, increased expression at 3 to 6 hr, and decreased expression at 24 hr. The expression of the genes in Cluster 9 is concentrated on the nasal surface.

Since no quantitative or positional changes were observed in key immune cells, such as *Ptprc, Cd4, Cd8a*, and *Cd19* (**Figure 2B**), we examined other cell types through integration of the ST profiles across the time series. Visualization of the spots through dimension reduction by Uniform Manifold Approximation and Projection (UMAP) analysis showed that our integration was successful, as a substantial fraction of the spots agreed across the time series (**Figure 4A**).

### Identification of S-CVP vaccine-induced genes in the NP region

**Figure 5:**
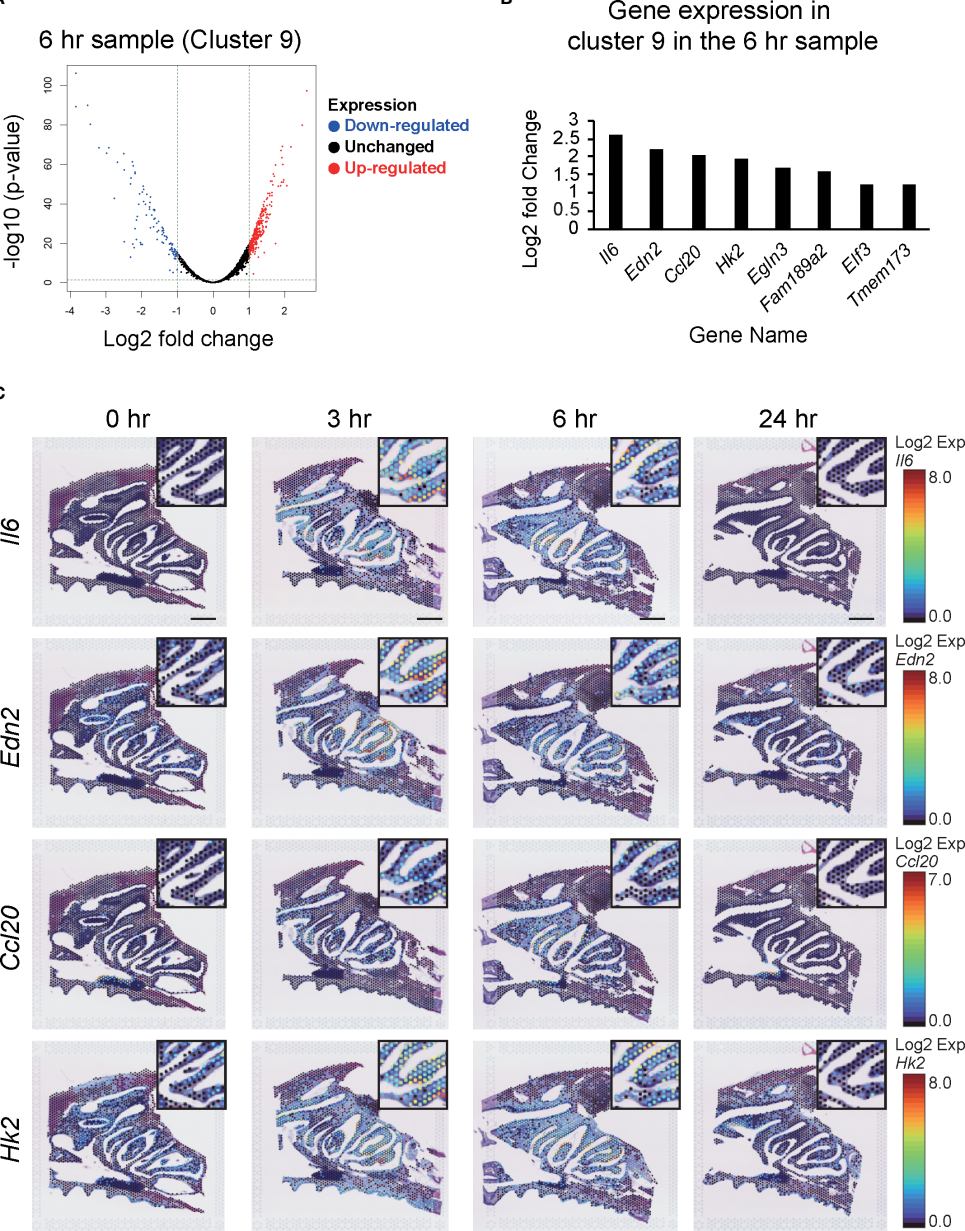
Changes in the expression of genes in Cluster 9 after S-CVP intranasal vaccination. **(A)** Differential analysis shown by a volcano plot showing Cluster 9 at 6 hr, with red dots showing upregulated genes and blue dots showing downregulated genes. **(B)** Genes showing similar patterns to *Il6* in Cluster 9 at 6 hr. **(C)** Spatiotemporal changes in *Il6*, *Edn2*, *Ccl20*, and *Hk2* expression in mouse nasal cavity at 0, 3, 6, and 24 hr. Each gene shows a characteristic pattern of low expression at 0 hr, increased expression at 3 to 6 hr, and decreased expression at 24 hr. Scale bar = 1 mm.

We also performed clustering of the spots based on their expression profiles. Notable exceptions were Clusters 6 and 9, which appeared only at 3–6 hr after vaccination, and then disappeared again at 24 hr (**Figure 4B**). In particular, Cluster 9 was restricted to the nasal cavity surface within the NP region. Analysis of the difference between the spots in Cluster 9 and the others at 6 hr post-vaccination showed that of the 3344 expressed genes, 295 were significantly upregulated (p values < 0.05 and log2 fold values ≥ 1) and 100 were downregulated (p value < 0.05 and log2 fold values ≤ -1) (**Figure 5A**). The change in expression levels of the genes with p values < 0.05 at 6 hr in Cluster 9 were ordered from the highest to the lowest.

The results showed that Interleukin-6 (*Il6*) had the highest log2 fold values, so we checked the site and time at which *Il6* was expressed using the Loupe Browser 6 program. The results showed a characteristic pattern in which *Il6* had the highest log2 fold values, with expression peaking at 3–6 hr and then decreasing at 24 hr. The genes that showed the same gene expression pattern (i.e., a small change in expression level at 0 hr, an increase at 3–6 hr, and a decrease at 24 hr) were then selected using the search function in the Loupe Browser 6 program. The results for genes with p values < 0.05 and those with log2 fold values ≥ 1 were sorted in descending order of log2 values. Among the genes with p values < 0.05 and log2 fold values ≥ 1, *Edn2* (Endothelin-2), *Ccl20* (C-C motif chemokine 20), *Hk2* (Hexokinase-2), *Egln3* (Prolyl hydroxylase EGLN3), *Fam189a2* (Endosomal transmembrane binding with Epsin1), *Elf3* (ETS-related transcription factor Elf-3), and *Tmem173* (Sting1) showed similar patterns to that observed for *Il6* (**Figure 5B**). In this study, we focused on the four genes with the highest log2 fold changes in the NP, i.e., *Il6*, *Edn2*, *Ccl20*, and *Hk2*, and examined their dot distribution patterns (**Figure 5C**).

### Identification of *Il6*, *Edn2*, *Ccl20*, and *Hk2* genes in DCs and macrophages

**Figure 6:**
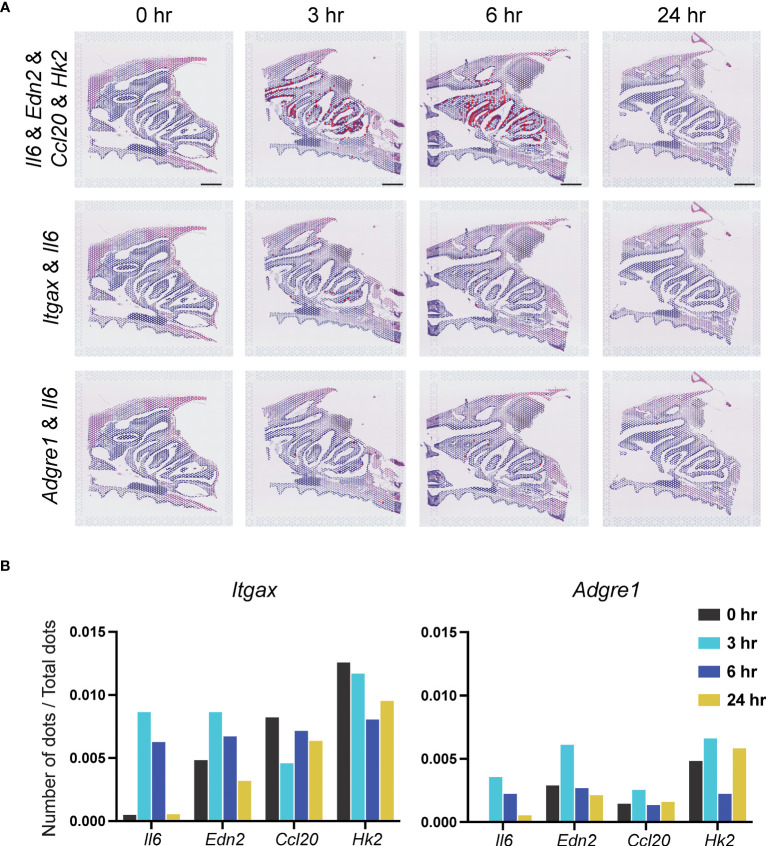
Identification of *Il6*, *Edn2*, *Ccl20*, and *Hk2* genes in dendritic cells and macrophages. **(A)** Top: Signal-positive dots indicating co-expression of *Il6*, *Edn2*, *Ccl20*, and *Hk2* genes at 0, 3, 6, and 24 hr. Scale bar = 1 mm. Middle and bottom: *Il6* and changes in expression at 0, 3, 6, and 24 hr in DCs and macrophages. **(B)** Number of signal-positive dots divided by the total number of dots is shown on the vertical axis. The values indicated by the bars indicate co-expression of the *Itgax* or *Adgre1* genes with *Il6*, *Edn2*, *Ccl20*, and *Hk2*.

The four genes in Cluster 9 in DCs (**Figure 6A**; **Supplemental Figure 4**), which are immune early response cells, were examined. At 3–6 hr after vaccination, an increase in positive cell signal dots for co-expression of the dendritic cell markers *Itgax* and *Il6* was observed in the NP region. However, *Edn2*, *Ccl20* and *Hk2* showed no change at 0, 3, 6, and 24 hr (**Figure 6B**).

A similar analysis was performed for macrophages (**Figure 6A**; **Supplemental Figure 5**). Positive cell signal dots indicating co-expression of the *Il6*, *Edn2*, *Ccl20*, and *Hk2* genes, as well as expression of the macrophage marker gene *Adgre1*, were observed in the NP region at 3–6 hr after vaccination. However, no significant changes in the number of signal-positive dots co-expressing the macrophage marker *Adgre1* and *Il6*, *Edn2*, *Ccl20*, and *Hk2* were observed. The values indicated by the bars indicate co-expression of the *Itgax* or *Adgre1* genes with *Il6*, *Edn2*, *Ccl20*, and *Hk2* (**Figure 6B**).

## General conclusion

In order to perform ST analysis in the mouse nasal cavity using the Visium system without compromising library sensitivity, the DV200 value had to exceed 50% after decalcification of the bone in the head. In this study, we were able to create eight tissue blocks with DV200 values of 79%, 85%, 78%, 82%, 78%, 74%, 78%, and 78% (**Supplemental Figure 1A**). We used a decalcification solution that contained both EDTA and citric acid, both of which are highly effective in decalcifying bone (30). As a result, we were able to decrease the decalcification time and may have been able to limit RNA degradation as a result. The gene expression profiles obtained by the ST analysis using the Visium system were consistent with morphological evaluations by HE staining (**Figures 1A, B**). Thus, we successfully established a protocol for Visium-based ST analysis in bone-containing tissues. We selected bone tissue because it is easy to identify and because we wanted to examine the effect of decalcification of the head. After selecting the bone region, many genes related to bone tissue and the surrounding muscles, such as *Col1a2* and *Bglap*, were identified as being among the 25 most expressed genes (**Figure 1A**). Thus, the morphological evaluation by HE staining and the gene expression results obtained by the Visium system were consistent. The findings showed that even with the decalcification process, ST analysis can be performed using the Visium system and that bone sections can be evaluated. To evaluate the immune response after intranasal CVP vaccination, we selected spots from areas that contained lymphocytes based on examinations of HE-stained cross-sections of a mouse head and gene expression analysis. The findings showed that genes related to olfaction, such as *Obp1a* and *Mup4*, were among the most expressed genes (**Figure 1B**). Furthermore, in selected spots, the Visium system was capable of detecting *Ptprc*, *Cd19*, *Cd4*, and *Cd8a*, which are representative of genes that are related to immunity; however, the expression of these genes was lower than that of genes related to olfaction (**Figure 1C**). Since lymphocytes cannot exist in the NP region without scaffold tissue, it can be inferred that the genes that are expressed by cells in the scaffold tissue are more highly expressed than those related to immunity.

Using established methods, the Visium ST analysis of mouse tissues after vaccination with the S-CVP intranasal vaccine showed that *Ptprc*, *Cd19, Cd4*, and *Cd8a*, which are widely known to be involved in immunity, were not only present in the NALT of lymphoid tissues, but also in the NP. No increase was observed in the number of spots or changes in the localization of *Ptprc*, *Cd19, Cd4*, and *Cd8a* at 3, 6, and 24 hr after S-CVP vaccination (**Figure 2B**). Since it typically takes several days to induce differentiation of these immunocompetent cells, further analysis is planned for several days after S-CVP vaccination.

M cells, which are specialized epithelial cells, transport foreign antigens into the lumen of the Peyer’s patches and nasal cavity by transcytosis. The antigen is passed to antigen-presenting cells, such as DCs, which are located in the vicinity of the M cells. These antigen-presenting cells then degrade the antigen, which activates T cells and B cells to produce secretory IgA and trigger mucosal immune responses (26–28). The Visium system can detect *Gp2, Sox8* and *Spib* genes in the nasal cavity (**Figure 3A**); these genes are representative of genes related to immunity in M cells (26–28). *Gp2-*, *Sox8-* and *Spib*-expressing cells were increased in the NP region at 3 and 6 hr after S-CVP vaccination, suggesting that M cells are activated in a short period of time (**Figure 3A**).

Cluster analysis of the cells in the tissue showed an increase in Cluster 6 and Cluster 9 in the NPs at 3–6 hr after vaccination with the S-CVP intranasal vaccine (**Figure 4B**). We focused on Cluster 9 because the expression of the genes in this cluster was concentrated on the nasal surface. Searching for gene expression patterns that were similar to those of *Il6*, we found that the expression of *Edn2*, *Ccl20*, *Hk2*, *Egln3*, *Fam189a2*, *Elf3* and *Tmem173* all increased in the nasal cavity at 3–6 hr (**Figure 5B**). Our findings showed that *Il6*, which had the highest log2 fold values of all of the genes within Cluster 9, as well as *Edn2*, *Ccl20*, and *Hk2*, all have a similar expression pattern (**Figure 5C**). In solid tumors, ET-2 (a protein encoded by the *Edn2* gene) is considered to contribute to the regulation of macrophage behavior and their distribution in hypoxic regions (31). We consider that S-CVP-vaccination might be involved in modulation of the inflammation microenvironment, with anti-viral immunity associated with the induction of ET-2 in this area. The cysteine–cysteine motif chemokine ligand 20 (CCL20) was up-regulated at 3–6 hr (**Figure 5C**). CCL20, which is also referred to as macrophage inflammatory protein 3α (MIP-3α) and liver- and activation-regulated chemokine (LARC), is the only ligand of CCR6 that is specifically expressed on immature DCs (32). CCL20 acts to recruit DCs to the inflammatory region. We also found that HK2 (a protein encoded by the *Hk2* gene) is up-regulated at 3–6 hr (**Figure 5C**). HK2 catalyzes the initial step of aerobic glycolysis that is essential for the proliferation of B cells as well as regulation of the effector function of T cells (33). The increased expression of HK2 may induce metabolic reprogramming that regulates the glycolytic system and activates immune cells.

**Figure 7:**
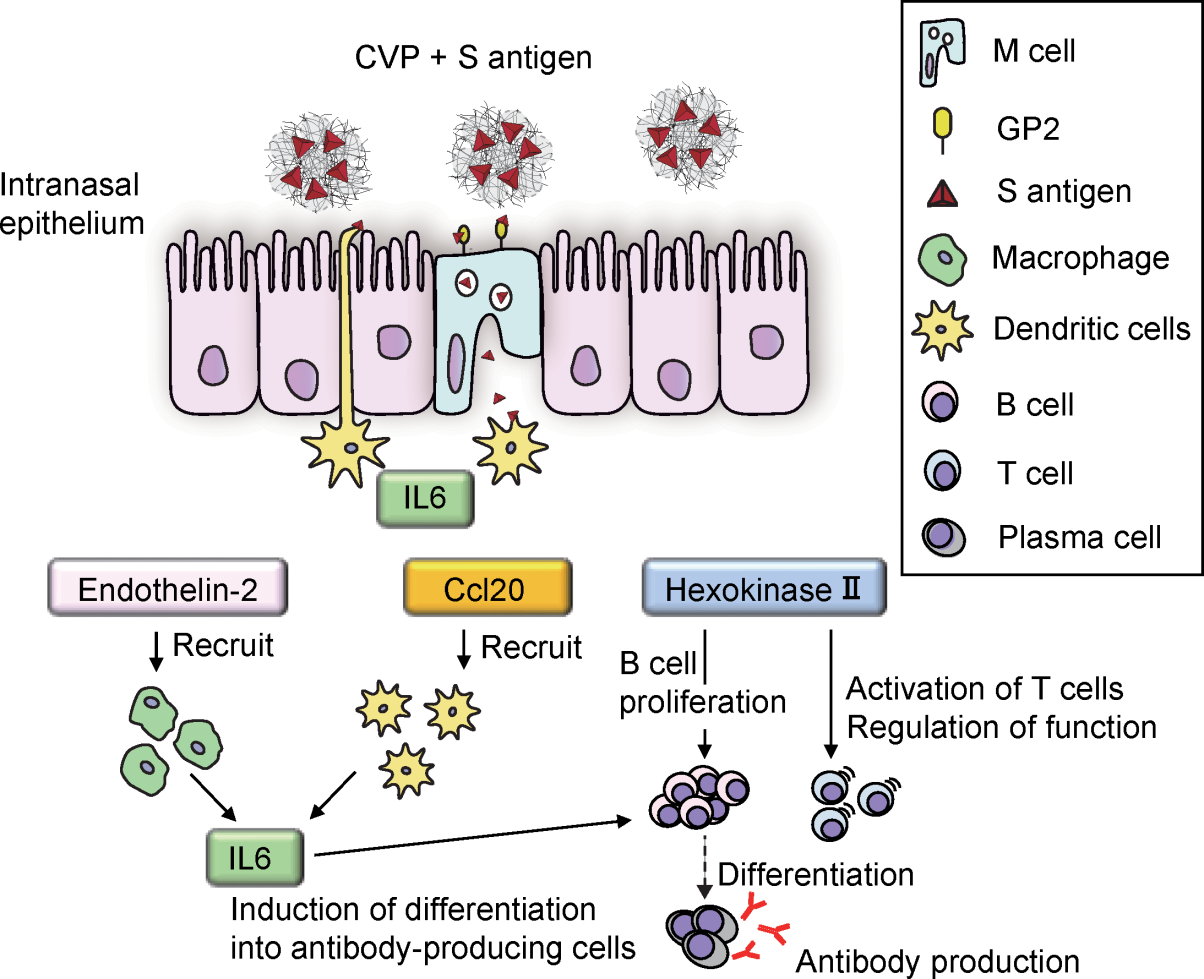
Outline of immunocompetent cell behavior shortly after S-CVP intranasal vaccination.

In this study, we used ST to analyze the changes in gene expression that contribute to the activation of M cells and immunocompetent cells after vaccination with antigen-CVP in the intranasal cavity of mice over time. As a result, a timeline could be produced showing the activation status and identity of the cell types involved in the initial induction of nasal mucosal immunity (**Figure 7**). Briefly, the following flow is suggested: 1) CVP and antigen mix to form large viscous particles with the antigen inside and on the surface; 2) the viscous nature of the particles increases their residence time in the nasal cavity; 3) CVP and antigen adhere to the M-cell surface owing to the viscous nature of these very large particles; given their large size, these particles are easily recognized by the cell membrane, which enhances their uptake into the M-cell; 4) the increased uptake of antigens into M cells leads to the production of cytokines, such as IL6; 5) cytokines such as IL6, which are produced by M cells, activate dendritic cells; and 6) cytokines, such as IL6, endothelin-2, CCL20, and hexokinase II, which are produced by activated dendritic cells then further activate dendritic cells, macrophages, and B cells. Further analysis of gene expression is expected to provide important fundamental data that can be applied to the development of highly effective mucosal vaccines.

In the future, using the ST method, it is expected that not only immunology, but also the mechanisms underlying the physiological phenomena that are specific to the nasal cavity, such as olfaction and allergic reactions, will be elucidated at the molecular level.

## Data availability statement

The data presented in the study are deposited in the DNA Data Bank of Japan (DDBJ) Sequence Read Archive (DRA) repository, accession number DRA016585 (https://ddbj.nig.ac.jp/resource/sra-submission/DRA016585).

## Ethics statement

The animal study was reviewed and approved by Tokyo Metropolitan Institute of Medical Science Animal Experimental Committee (Approval No. 22-078).

## Author contributions

ST, TH, TM, and MK conceived the study. ST, TH, SI, SH, KY, YT, TM, and MK carried out the experiments. MK supervised the study. ST, TH, SI, SH, KY, YM, HK, TM, YK, and MK participated in data analysis, interpretation, and manuscript review. ST, TH, SI, SH, YM, TM, and MK wrote the manuscript. All authors contributed to the article and approved the submitted version.
