# Supplementary material for: Application of spatial transcriptomics analysis using the Visium system for the mouse nasal cavity after intranasal vaccination
Source: Front Immunol. 2023 Jul 21;14:1209945. doi: 10.3389/fimmu.2023.1209945 (PMC10403337; doi:10.3389/fimmu.2023.1209945)
Supplement: Supplementary file 1 [file DataSheet_1.pdf]

Initial behavior of immunocompetent cells during intranasal vaccination inferred by spatial transcriptomics

Sakiko Toyama[1,2], Tomoko Honda[1], Sadahiro Iwabuchi[3], Shinichi Hashimoto[3], Kenzaburo Yamaji[1], Yuko Tokunaga[1], Yusuke Matsumoto[1,7], Hideya Kawaji[4], Takashi Miyazaki[5], Yoshiaki Kikkawa[2,6], Michinori Kohara[1]*

*** Correspondence:** Michinori Kohara, E-mail: kohara-mc@igakuken.or.jp

Supplemental Figures

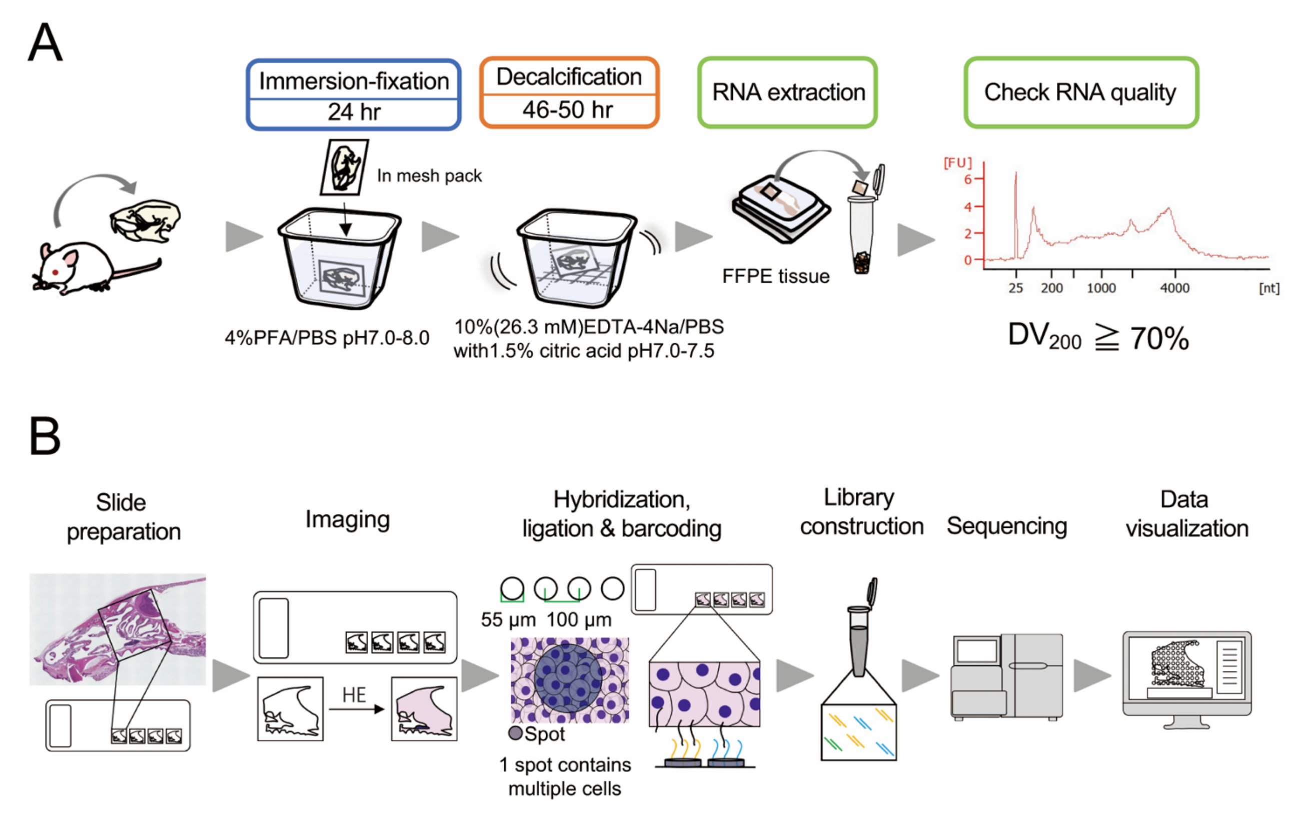

Supplemental Figure 1. Application of the Visium system to assay gene expression in mouse nasal tissues.

(A) Schematic diagram showing the preparation of FFPE blocks containing bone tissue for spatial transcriptome analysis using the Visium system.

(B) Spatial transcriptome workflow using the Visium system.

FFPE tissues are placed onto a Visium Spatial Gene Expression slide, stained with hematoxylin and eosin, and images of the tissue sections are prepared. The glass slide captures mRNA with barcoded oligonucleotide probes and cDNA synthesis is followed by library construction. Whole-transcriptome gene expression libraries are prepared using Illumina instruments. The gene location of expression is correlated with tissue morphology using Space Ranger and Loupe Browser 6 software from 10x Genomics.

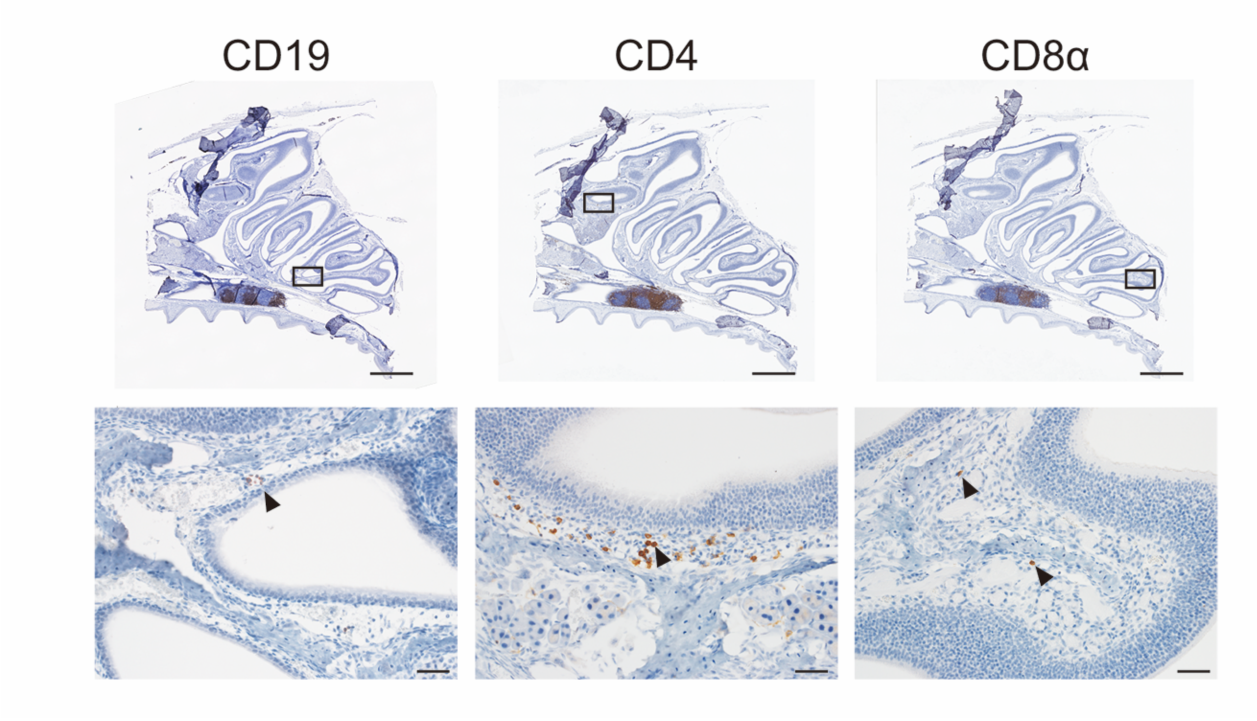

Supplemental Figure 2. Immunohistochemical staining for CD19, CD4, and CD8α in naïve mouse sample.

Immunohistochemical staining images (Left: CD19, middle: CD4, right: CD8α) are shown. The upper panels show the whole view of the nasal passages. The lower panels are high magnification images of the black insets in the image above.

Arrowheads indicate positive cells.

Top row: Scale bar = 1 mm

Bottom row: Scale bar = 50 µm

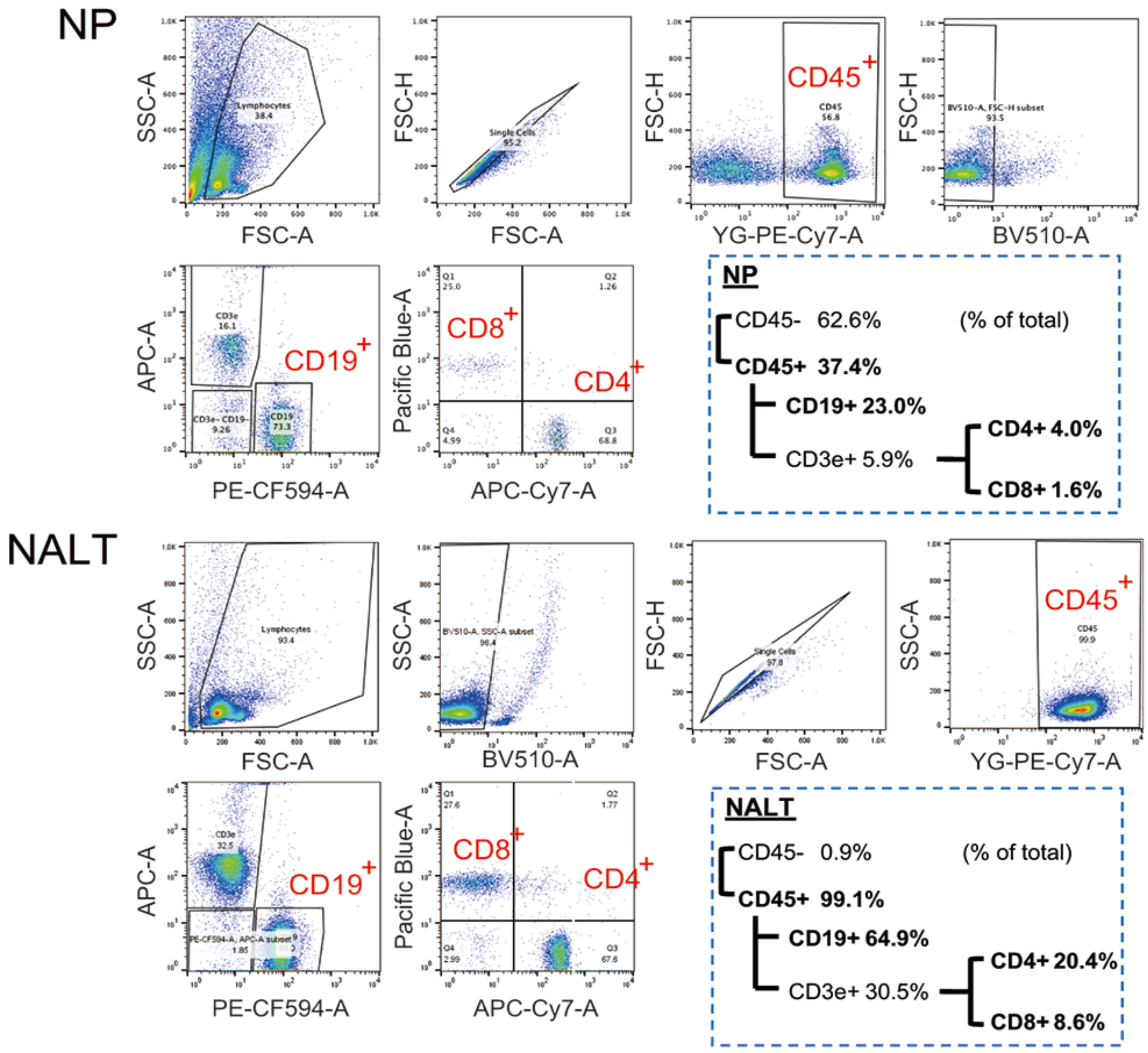

Supplemental Figure 3. FACS analysis data for nasal passages (NP) and nasal-associated lymphoid tissue (NALT).

Lymphoid cells in the NP and NALT of the mice were isolated. The number of CD45+, CD19+, CD3e+, CD4+ and CD8+ cells in the NP and NALT was then calculated based on the total number of lymphoid cells in the NP and NALT and the frequency of this subset in the population (as determined by FACS analysis). Results of fluorescence activated cell sorter (FACS) analysis are representative results of six independent experiments are shown. The values in the square indicate the average percentage of six independent experiments.

Top: FACS analysis data from the NP region, Bottom: FACS analytical data from NALT.

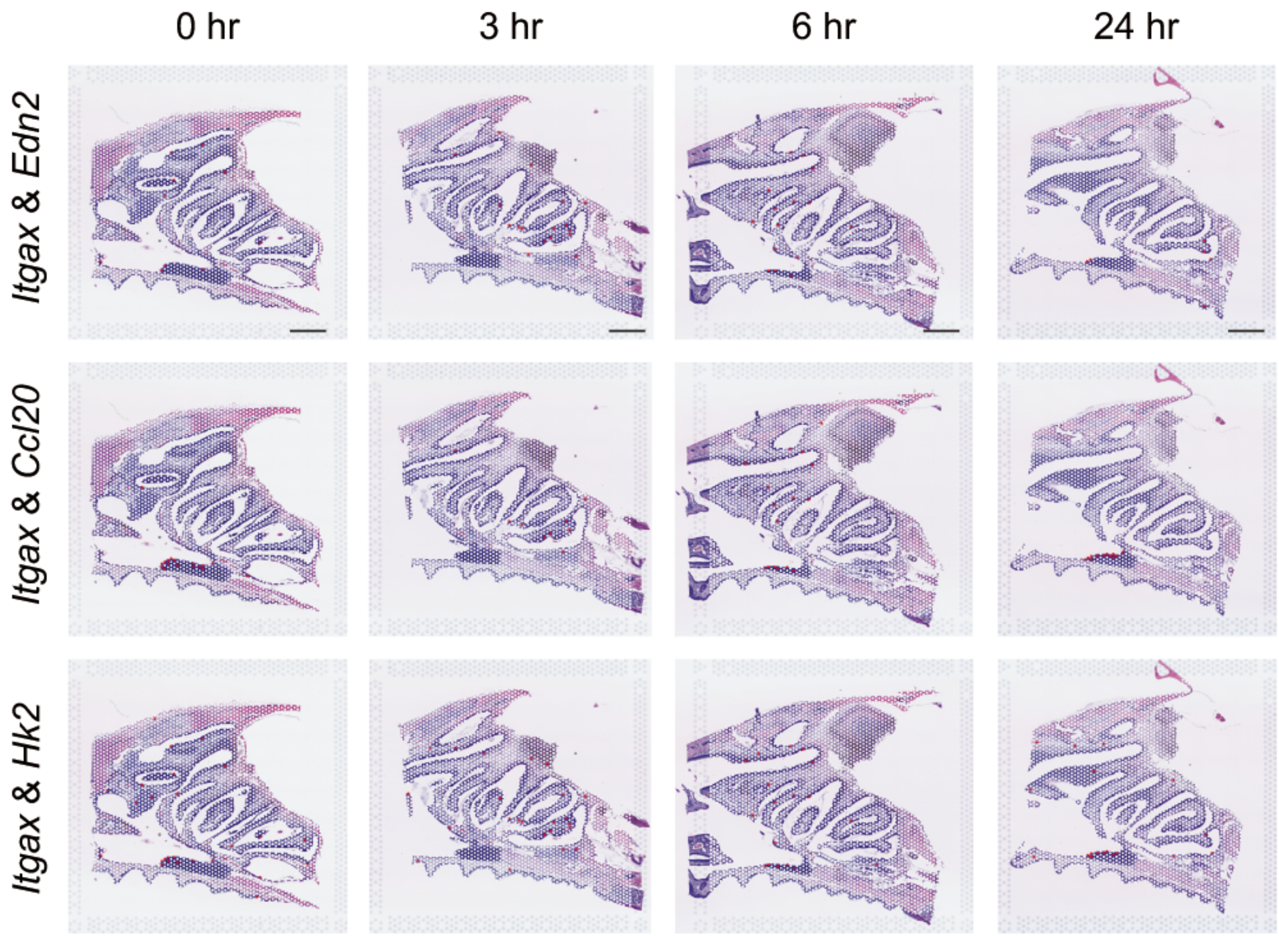

Supplemental Figure 4. Identification of *Edn2*, *Ccl20*, and *Hk2* genes in dendritic cells.

The three most expressed genes in Cluster 9 and changes in their expression at 0, 3, 6, and 24 hr in dendritic cells.

Scale bar = 1 mm

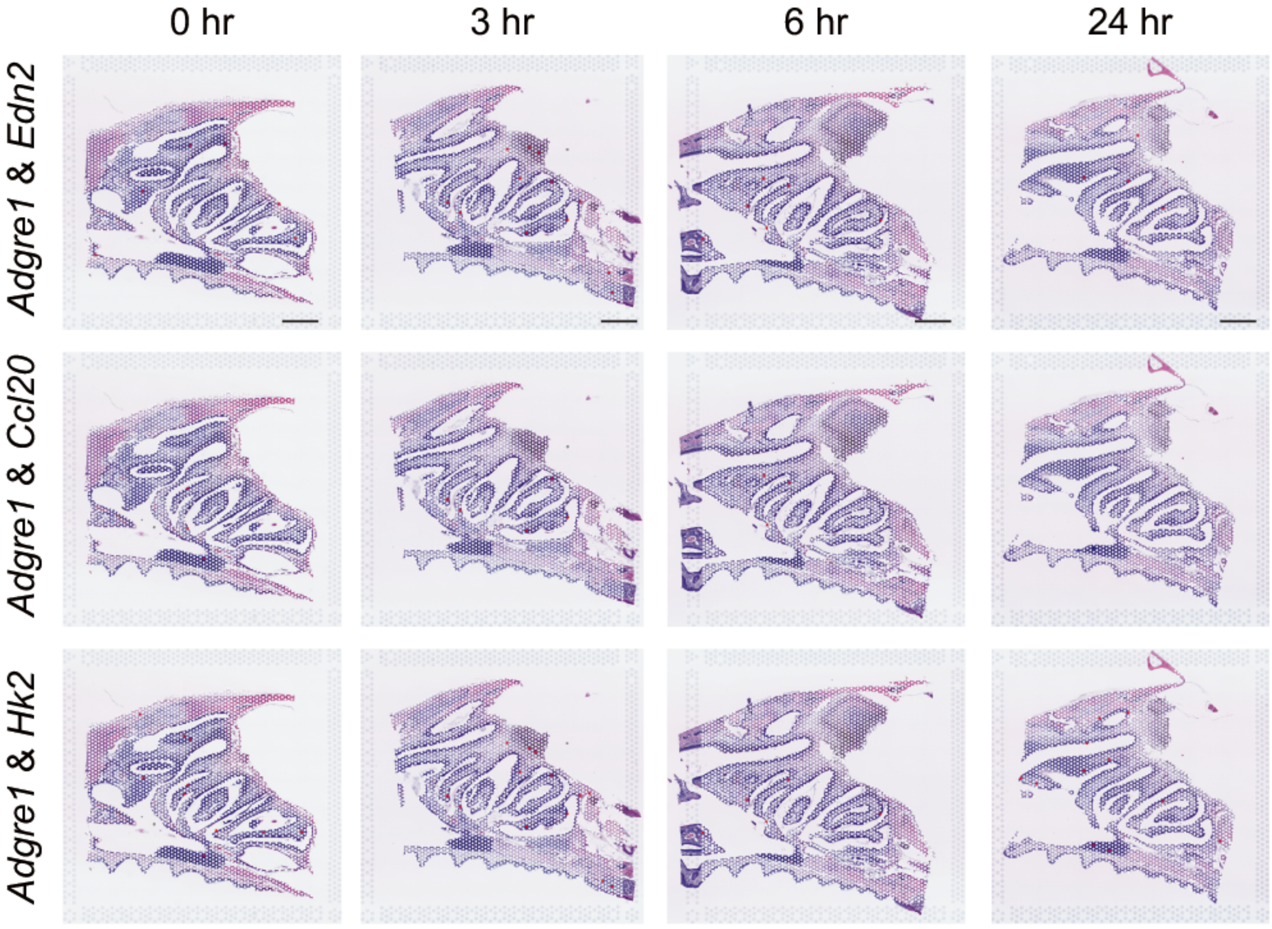

Supplemental Figure 5. Identification of *Edn2*, *Ccl20*, and *Hk2* genes in macrophages.

The three most expressed genes in Cluster 9 and changes in their expression at 0, 3, 6, and 24 hr in macrophages.

Scale bar = 1 mm
